# Short- and long-distance transport: health, survival and growth of preweaned dairy and dairy-beef cross calves

**DOI:** 10.1093/jas/skaf341

**Published:** 2025-09-29

**Authors:** Gustavo M Schuenemann, Juan M Piñeiro

**Affiliations:** Department of Veterinary Preventive Medicine, The Ohio State University, Columbus, OH 43210; Department of Animal Sciences, Texas A&M University, College Station, TX 77843

**Keywords:** calf transport duration, health, growth, survival

## Abstract

The objective of this retrospective, observational study, was to assess the association of transport duration (0.5, 8, 17, or 24 h) with calf survival, diseases (diarrhea and pneumonia), and preweaning average daily gain (ADG). A total of 392,064 calves (dairy females = 125,901 transported 0.5 or 24 h and dairy-beef [DB] cross = 266,111 transported 8 or 17 h) born from Holstein-Jersey (HxJ) dams at 15 farms under the same overall management were included (from January 2022 through March 2024). Calves (female = 146,163 and male = 125,953) were transported following the conditioning protocol in 2,973 loads with a mean age 3.2 d to two separate calf raising-facilities. Dystocic births, failure of passive transfer (FPT) of immunity, calf birth and weaning weights, birth season, calf sex, calf breed, and birth year were recorded. Calf diarrhea and pneumonia, mortality upon arrival at calf raising-facility and up to weaning (60 ± 3 d), and ADG from birth to weaning were recorded. Data were analyzed using GLIMMIX, MIXED, PHREG, or PROC LOGISTIC procedures of SAS. Dam parity, calf health, calf birth season and year, FPT, dystocia, calf sex, calf breed and calf age at transportation were associated with calf mortality up to weaning (*P *< 0.05). Overall, calf mortality upon arrival at the calf-raising facility was 0.015%, and it did not differ statistically by transport duration. Calves fed two colostrum meals had less FPT compared to one meal (*P *< 0.0001), regardless of sex or breed. Overall, calf mortality at weaning was 2.49% but varied by transport duration with 3.56% (0.5 h), 1.01% (8 h), 2.18% (17 h), and 1.55% (24 h; *P *< 0.0001). Calf mortality at weaning differed (*P *< 0.0001) by transport duration, mostly due to FPT, calf diseases (pneumonia and diarrhea), female dairy calves born to first-calf heifers, sex within DB, birth season, birth year, calf-raising facility and gestation length. Dairy calves transported 24 h (0.76 ± 0.01 kg/d) had higher ADG compared to those dairy calves transported 0.5 h (0.65 ± 0.02 kg/d; *P *< 0.0001); primarily due to the confounding effect of parity. ADG did not differ for DB calves transported 8 h (0.86 ± 0.04 kg/d) or 17 h (0.82 ± 0.02 kg/d), regardless of parity. While a causal relationship between transport duration and survival or ADG cannot be established, these findings show that key health-related factors early in life play a much larger role in calf mortality at weaning than transport duration.

## Teaser

This study provides new insights on the relationship of transport duration with health, survival, and growth of preweaned dairy and dairy-beef cross calves. Findings highlight that key health-related factors early in life play a much larger role in calf mortality at weaning than transport duration.

## Introduction

For any dairy operation, calving is an essential requirement of the production system in which cows initiate lactation and provide the future replacements of the herds as well as nonreplacements (male and female). In the US, over 90% of nonreplacement calves are raised off-site; thus, requiring transportation to move young calves to complete their growing phase (USDA, 2016). For replacement heifers, about 50% of dairy operations had heifers born on-site but were raised off-site with an average transport distance of 80 km, ranging from 14 to 800 or more km (USDA, 2016). The transportation of preweaned dairy calves has triggered much debate recently due to welfare concerns directly associated with their health status, survival, and overall performance (Cramer et al., 2024). It has been shown that young calves are more susceptible to diarrhea <14 d of age due to failure of passive transfer of immunity (FPT; Raboisson et al., 2013) and other stressors (e.g., restricted access to water and milk, comingling in large groups, exposure to increased temperature-humidity index [THI]) mostly due to their undeveloped immune system and physiological response (Hulbert and Moisá, 2016).

Much of the recent discussion has been focused on the age of young calves at transportation, colostrum management, and FPT. Although replacement heifers are often transported at an average age of 3 d (USDA, 2016), nonreplacement calves are typically transported within 24 h after birth to <1 wk of age (Shivley et al., 2019; Cramer et al., 2024). When it comes to preweaned calf transportation, most of the studies reported data using veal calf facilities (Pempek et al., 2017; Scott et al., 2019; England et al., 2023) and to a lesser extent non-veal calf facilities raising nonreplacement calves (Pempek et al., 2023; Cramer et al., 2024). It has been shown that poor colostrum management almost always resulted in greater FPT with the subsequent increment of calf morbidity and mortality preweaning (Lombard et al., 2020). However, it is also known that dairy calves born from short gestation dams (255–269 d) are more likely to be stillborn and experience more health events early in life compared to average gestation length (270–283 d; Vieira-Neto et al., 2017). There is limited or no information available in the literature comparing the effect of calf transport duration relative to calf type (dairy female calves vs dairy-beef [DB] cross males or females) born within two distinct geographical regions (Southwest and upper Midwest) but under the same management from birth to weaning. Therefore, the objective of the present retrospective, observational study was to assess the association of transport duration (0.5, 8, 17 or 24 h) with calf survival, diseases (diarrhea and pneumonia), and preweaning average daily gain (ADG). We hypothesized that calves exposed to transport longer than 8 h will have increased incidence of diarrhea and pneumonia, decreased survival, and reduced preweaning ADG.

## Materials and Methods

This retrospective observational study was conducted from January 2022 through March 2024. Since this was an observational study and data points were collected through existing on-farm records, the study protocol was determined to be exempt from review by The Ohio State University Institutional Animal Care and Use Committee.

### Experimental design and sample size calculation

This is a retrospective observational study with data collected from one closed dairy production system composed of 15 dairy farms under the same overall management. Each individual calf was tracked from birth to weaning, and information on gestational factors such as gestation length was also collected, to assess the impact of transport duration on calf survival while carefully controlling for potential confounders. It is important to note that this was an observational study and, as such, while we made efforts to adjust for confounding factors, statistical significances and causal relationships should be interpreted with caution. A retrospective sample size calculation was performed to be able to detect a difference in preweaning calf mortality of 0.5 percentage points (from 2 to 2.5% with SD of 10%) following transportation (duration 0.5 versus 24 h) with adequate statistical power (1−β = 0.8) and statistical significance (alpha = 0.05), a sample size of 6,281 calves per group were required (POWER procedure of SAS; SAS Institute Inc., Cary, NC).

### Animals and facilities

**Figure 1.**
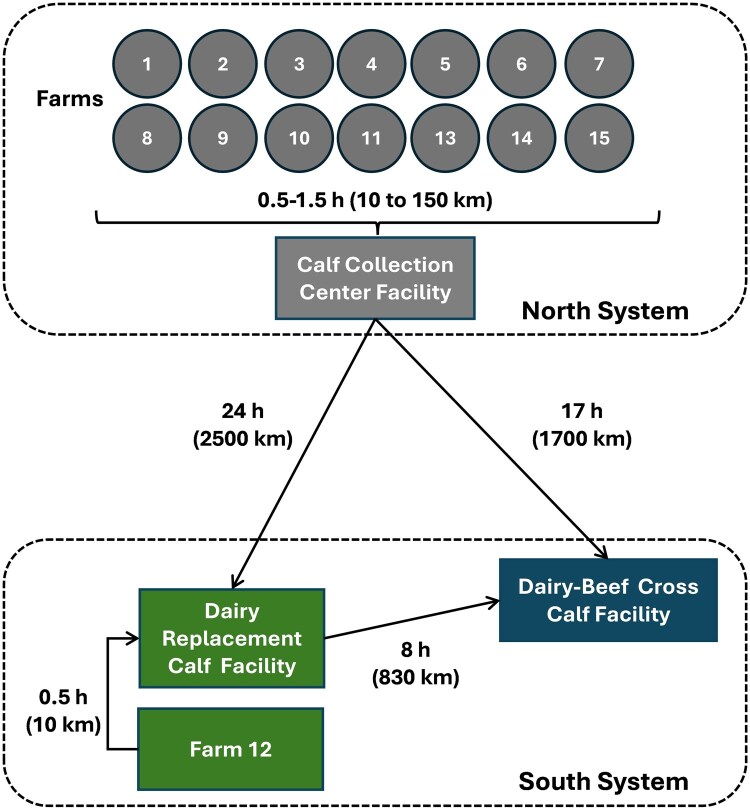
Schematic representation showing locations of dairy farms with maternities, calf collection center and calf transport duration to two calf-raising facilities. In the north system (Minnesota and South Dakota), there were a total of 14 dairy farms (each with their own maternity) and a calf collection center facility. In the south system, one dairy farm housed all first-calf heifers with its own maternity (farm 12; Arizona). Calves were transported and raised in two separate facilities with similar housing and environmental conditions; one located in Arizona for replacement heifers transported 0.5 (*n* = 122,407) and 24 h (*n* = 37,117), and another in New Mexico for DB calves (males and females) transported 8 (*n* = 20,485) and 17 h (*n* = 212,055). For calf transport lasting 17 and 24 h, each trailer was operated by a team of two drivers. Arrows indicates the direction for calf transport duration of 0.5, 8, 17, or 24 h (10, 830, 1700, or 2500 km, respectively).

All animals and facilities described below are part of a single, integrated production system, which consists of multiple dairy farms with the same genetic base for cows and two calf-raising facilities, all operated under the same overall management and transport logistics. This cohesive setting allowed for tracking of each individual calf from birth to weaning. A total of 392,064 calves born from Holstein-Jersey (HxJ) cross dams (average live body weight of ∼510 kg) from 15 dairy farms were enrolled in this retrospective observational study. All dairy farms were under the same management and divided into two systems according to their geographical US locations: 1) North system consisted of 14 dairy farms located in South Dakota and Minnesota (*n* = 249,172 calves) and 2) South system located in the Southwest (Arizona and New Mexico) with 1 dairy farm for all first-calf heifers (*n* = 142,891 calves; Figure 1). All farms combined had an overall stillbirth of 1.5%, 21-d pregnancy rate of 31% with 4.5% total abortions, and ECM of 35 kg/d (3.8% milk protein and 5.1% milk fat) with SCC of ∼120,000 cells/mL. Dairy replacement heifers were born from HxJ dams bred with dairy sires using sex-sorted semen and DB cross calves were born from HxJ dams bred with beef sires (Limousin) using conventional semen. In the north systems, all cows were housed in cross-ventilated barns with freestalls bedded with recycled manure from digesters (∼38% dry matter basis). In the south system, all first-calf heifers were housed in dry-lots with air-free movement roof structure covering the central feedbunk and bedding area on both sides. All calves were shipped in 2,973 loads to two separate, but similar calf-raising facilities located in the Southwest, one for nonreplacement calves (male and female) located in New Mexico and one for all replacement heifers located in Arizona. Upon arrival and regardless of farm of origin, all calves were housed into individual hutches up to weaning (60 ± 3 d of age) at both calf-raising facilities.

### Management of prepartum dams and maternity

All prepartum cows and first-calf heifers were fed twice daily, in the morning and afternoon. In addition, dairy heifers and cows were fed a total mixed ration (TMR) to meet or exceed nutritional requirements (NASEM, 2021; Table 1). Pregnant cows (Lactation ≥1; cows) were dried off 48 ± 3 d prior to expected calving date and moved into the far-off dry pen immediately after last milking. Both multiparous and first-calf pregnant heifers (Lactation = 0) were moved into prepartum pens at 21 d prior to expected calving date.

**Table 1.** Ingredients and nutrient composition of typical formulated far-off and prepartum diets (DM basis)

	South system		North system	
Ingredients, % of DM	Far-Off	Prepartum	Far-Off	Prepartum
**Wheat straw**	–	–	43.93	30.02
**Corn silage**	19.23	29.16	33.26	43.56
**Wheatlage**	64.69	55.74	–	–
**Beet pulp pellet**	–	–	–	7.27
**Soybean meal**	9.92	–	18.71	7.01
**Amino plus**	–	6.67	–	1.98
**Dry distillers**	5.12	1.78	2.00	1.98
**Wet gluten**	–	–	–	1.98
**AA blend**	–	2.16	–	1.58
**Calcium carbonate**	–	1.22	1.16	1.07
**Anion mineral supplement**	–	1.78	–	2.34
**Mineral and vitamins mix**	1.05	1.44	0.94	1.20
**Molasses-liquid**	–	0.05	–	–
**Nutrient profile, % of DM unless otherwise noted**				
**NE_L_, Mcal/kg**	1.43	1.52	1.41	1.45
**CP**	14.17	15.15	14.95	14.10
**NDF**	41.15	37.29	45.63	43.87
**Forage NDF**	38.93	36.04	43.31	38.10
**Starch**	13.98	16.34	12.33	16.76
**Sugar**	5.76	6.11	4.06	4.09
**EE**	3.61	3.51	2.46	2.92
**Ca**	0.49	0.75	0.75	0.75
**P**	0.34	0.32	0.25	0.25
**Mg**	0.20	0.50	0.30	0.50
**K**	1.52	1.56	1.57	1.27
**Na**	0.13	0.10	0.10	0.10
**Cl**	0.69	1.00	0.50	0.95
**S**	0.21	0.28	0.23	0.33
**DACD, mEq/kg DM^1^**	122.85	−140.82	162.5	−102.16

1 Dietary cation–anion difference (DCAD) was calculated as follows: DCAD = (mEq of Na + mEq of K) − (mEq of S + mEq of Cl).

All cows were moved into an adjacent individual maternity pen for parturition and first-calf heifers calved in the dry-lot pen. The calving ease of cows (assistance provided at birth) was recorded by on-farm personnel using a 4-point scale (1 = no assistance provided; 2 = light assistance by one person without the use of mechanical traction; 3 = mechanical extraction of the calf with an obstetric calf-puller; and 4 = severe dystocia: surgery or fetotomy needed; Schuenemann et al., 2011). Assisted calving was defined as a dam with a score of ≥2 at calving using a 4-point scale.

The gestation length of prepartum HxJ cross heifers and cows were classified as short (mean = 262 d with ±1 SD; ranged from 254 to 269 d), average (mean = 276 d with a population mean ± 1 SD; ranged from 270 to 283 d) or extended (mean = 290 d with ±1 SD; ranged from 284 to 298 d). Calf birth date, calf sex (male or female), and calf breed (dairy and DB cross) were recorded. After calving, cows were milked to harvest colostrum and moved into the postpartum pen, and calves were moved into maternity pens for further processing. All live born calves were washed with warm water, identified with ear tags, birthweight recorded using a digital scale, and the navel was disinfected with NavelDyne solution within 2 h after birth. All calves (female = 146,163 and male = 125,953) were fed 3.8 L of colostrum with ≥23% Brix within 2 h of birth and calves born in the north system were fed a second 2 L colostrum feeding 6 h later. The conditioning protocol prior to transportation consisted of 4 consecutive feedings (each of 2 L per calf, the fourth milk feeding was mixed with 1.9 L electrolyte). All calves that drank 4 consecutive meals and without experiencing any signs of sickness were considered fit to transport. Only calves with dried umbilical cord were shipped out of the maternity. Newborn calves in Arizona were moved from maternity to individual hutches within 24 h after birth, while those calves born in the north system were transported within 24 h of age to a nearby collection center (Figure 1).

### Calf transport and trailer measurements

**Figure 2.**
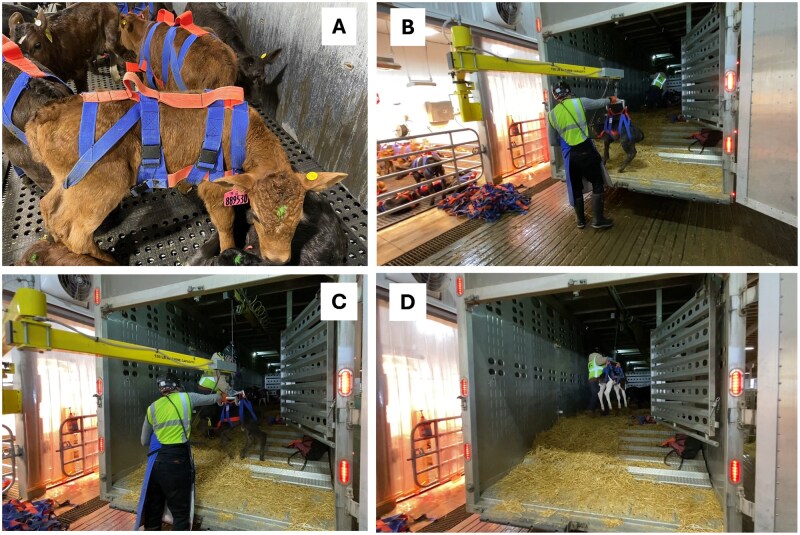
Schematic representation describing the process of loading calves in and out of trailers at the maternity and at the collection center, respectively, within the current study. Each individual calf was fitted with a strap harness (A). Once the trailer arrived at the maternity, an operator loaded each calf individually using a Conco^®^ articulating arm lifter fitted with a winch connected to a hook (B). Transportation trailers were also fitted with rail mounted inside the roof with a moving winch connected to a hook (C). The hook fitted to the outside lifter arm was wider than the hook fitted to the trailer allowing to gently handle the calf in and out of the trailer (D). At least two operators fitted with protective equipment (e.g., helmets) worked as a team, one inside the trailer and one outside, to load and unload calves. Handling and safety for both calves and operators were top priorities. These images were created by the authors.

In the north system, all newborn calves were shipped once per day from each farm of origin (0.5 to 1.5 h away) to a central calf-facility with individual hutches bedded with deep wheat straw inside a naturally ventilated barn with supplemental tube ventilation for conditioning prior to transportation (17 or 24 h) to the southwest calf raising-facilities (Figure 1). Calves were transported to two separate calf-raising facilities with similar housing and environmental conditions; one located in Arizona for replacement heifers transported 0.5 (*n* = 122,407) and 24 h (*n* = 37,117), and another located in New Mexico for DB calves (male and female) transported 8 (*n* = 20,485) and 17 h (*n* = 212,055). The estimated transport distance was 10, 830, 1700, and 2500 km for calves transported 0.5, 8, 17 or 24 h, respectively. For calf transport lasting 17 and 24 h, each trailer was operated by a team of two drivers with two short stops. All calves were transported in a single continuous drive without unloading. In the south system, all calves born were shipped daily within 24 h from the maternity to a nearby (0.5 h) calf-raising facility (individual hutches) and some nonreplacement calves were shipped to another calf-raising facility 8 h away at 6 ± 3 d of age (individual hutches). The process of loading calves in and out of trailers at the maternity and at the collection center, respectively, within the current study is described in Figure 2. Briefly, each individual calf was fitted with a strap harness at the maternity. Once the trailer arrived at the maternity, an operator loaded each calf individually using a Conco^®^ articulating arm lifter fitted with a winch connected to a hook (Positech Corporation, Laurens, IA). Transportation trailers were also fitted with a rail mounted inside the trailer roof with a moving winch connected to a hook (Positech Corporation, Laurens, IA). The hook fitted to the outside lifter arm was wider than the hook fitted to the trailer allowing gentle handling of the calf in and out of the trailer. At least two operators fitted with protective equipment (e.g., helmets) worked as a team, one inside the trailer and one outside, to load and unload calves. Handling and safety for both calves and operators were top priorities.

**Figure 3.**
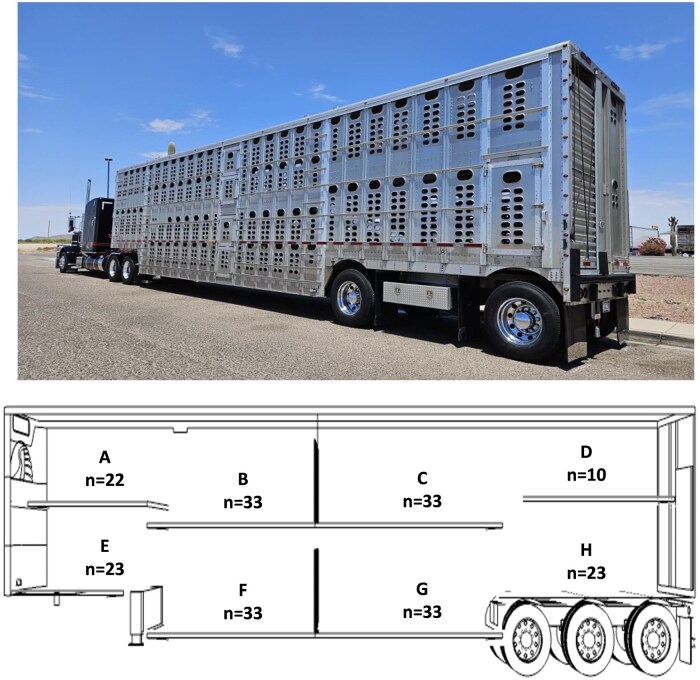
Schematic representation of compartments for a two-deck trailer used to transport calves (*n* = 210) within the current study. Actual trailers had either 2 or 3 rear axles. For calves transported 8 or more h, an average of 210 calves were loaded inside a two-deck trailer (∼75.66 m^2^), with each calf allotted ∼0.36 m^2^ of space, and bedded with sawdust. Sections A and E is the nose with 22 and 23 calves, respectively. Sections B and C is the deck with 33 calves each (66 calves total). Sections F and G is the belly with 33 calves each (66 calves total). Section D is the doghouse with 10 calves. Section H is the back with 23 calves. Both images are original and were provided by the participating dairy farm, used with permission.

For all live calves transported from 0.5 to 1.5 h, groups of 50 to 100 calves were transported in 1,786 loads using either gooseneck (∼15.60 m^2^) or single deck trailers (42.36 m^2^), both bedded with wheat straw. This provided ∼0.31 to 0.42 m^2^ of space per calf, respectively. For live calves transported 8 or more hours, an average of 210 calves in 1,187 loads (±44, representing 1 SD) were loaded inside a two-deck trailer (∼75.66 m^2^), with each calf allotted ∼0.36 m^2^ of space, and the trailer was bedded with sawdust (Figure 3). All trailers used for calf transportation of ≥8 h were thoroughly washed and disinfected after transportation. Trailers used for short-distance transportation were thoroughly washed and disinfected once per day.

### Calf fit-for-transport criteria

In the north systems, all live born calves that were fed colostrum and had their umbilical cord dried were transported (0.5 to 1.5 h) to a nearby central collection center once per day at 24 h of age. At the central collection facility, calves were housed in individual hutches inside a large naturally ventilated with supplemental tube ventilation barn and fed 4 L per day of milk replacer with 14% solids (2 L per feeding per calf, twice per d). The conditioning protocol prior to transportation consisted of at least four consecutive feedings: each of 2 L per calf and the fourth milk feeding was followed by 1.9 L electrolyte (Land O’Lakes^®^ electrolyte base, Land O’Lakes Inc., Saint Paul, MN). All calves who drank four consecutive meals at the collection center facility and without experiencing any signs of sickness were considered fit to transport starting at 3 d of age. Calves were loaded onto the trailers between 30 min and 1 h after their last feeding. All transported calves from the north system were raised using individual hutches bedded with deep corn stalks until weaning (60 ± 3 d of age). In the south system, live newborn calves were fed colostrum and transported within 24 h of birth to a nearby calf-raising facility with individual hutches bedded with deep corn stalks. Dairy replacement heifers stayed until weaning (60 ± 3 d of age) and nonreplacement DB cross calves were transported (8 h) to another calf-raising facility at 6 ± 3 d of age until weaning (60 ± 3 d of age).

### Housing and feeding program for pre-weaned calves

Preweaned calves were housed in individual plastic hutches bedded with deep corn stalks and had access to an outside patio enclosed with a metal fence located in front of each hutch. All calves were fed 1.9 L of milk twice per day from arrival at the calf-raising facilities through approximately 20 d of age and then they were fed 2.84 L of milk twice per day from 21 d through approximately 46 d old. Weaning then consisted of 1 week of feeding 2.84 L once per day, followed by 1 week of feeding 1.89 L once per day until fully weaned at approximately 60 ± 3 d old. The milk diet consisted of either non-fat dry milk or condensed skim milk, liquid fat (primarily tallow with added coconut oil) and a VTM (vitamin-trace mineral) pack. Milk was formulated for approximately 27% protein and 26% fat for calves 20 d old and less and for approximately 28% protein and 24% fat for calves over 20 d old. Percent solids of the milk diet ranged from 13.5% to 15.25%. A textured starter diet composed of whole corn, a protein pellet and molasses was fed ad libitum throughout the entire hutch phase. The starter formula for calves less than 60 d old was approximately 19% crude protein and 35% starch on an as-fed basis. The starter formula for calves 60 ± 3 d old and over was approximately 17% crude protein and 35% starch on an as-fed basis. Each individual calf had ad libitum clean drinking water in a plastic bucket with a maximum capacity of 7.5 L. All preweaning calves received the same nutrition program and underwent identical health screening and treatments.

### Assessment of calf passive transfer of immunity, health status, survival, and growth preweaning

All preweaned calf health, survival and performance data were obtained from on-farm computer records (DairyComp 305, Valley Agricultural Software, Tulare, CA). Pregnant dams with gestation length shorter and longer than 3 SD from the mean were removed for data analyses (Vieira-Neto et al., 2017). FPT was assessed in a subset of calves (*n* = 46,377) and defined as the total calf serum protein <5.1 mg/dL at 2–3 d of age (Godden et al., 2019; Lombard et al., 2020). Calf mortality upon arrival was defined as a calf that died upon arrival at calf-raising facility following transportation. While the date of death was recorded for any calf that died, it was not possible to determine if death occurred during transport or immediately upon arrival (e.g., shortly after unloading). Calf mortality up to weaning was defined as a calf that died upon arrival at calf-raising facilities and up to weaning at 60 ± 3 d of age. Farm personnel from both calf-raising facilities screened and recorded all health events daily using a fecal and respiratory scoring system. A case of calf diarrhea was defined as a watery fecal discharge (score 4 using a 4-point scale; Larson et al., 1977). A case of calf pneumonia was defined as the presence of abnormal ocular and nasal discharge, cough, and high rectal temperature (score ≥5 using the Wisconsin calf respiratory scoring chart; Buczinski et al., 2015). Calf birthweights were recorded at calving for all calves, and in a subset of calves (*n* = 84,449), their live body weights (BW) were recorded after weaning in groups. Individual ADG was calculated by subtracting calf birthweights from group weaning BW and then dividing by their age (d) at weaning, which was standardized to 60 d to adjust for age differences.

### Statistical analyses

Data from individual calves born (e.g., birth date, parity, dystocia scoring, sex, colostrum, transport duration, health status, survival) were exported from DairyComp 305 into an Excel spreadsheet. Outliers were carefully investigated for each model using scatter plots and studentized residuals to avoid potential leverage or influential points. The Shapiro-Wilk test statistic histograms and Q-Q plots were used to assess normality of the residuals once obtained from their respective final models.

#### Demographic distribution of newborn calves

**Figure 4.**
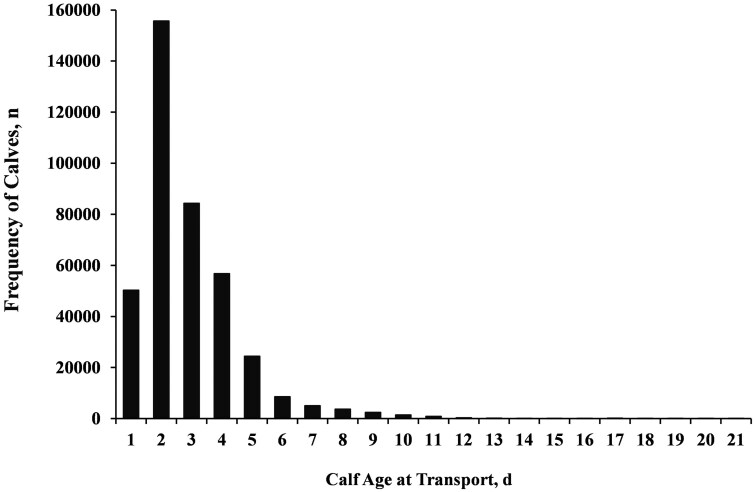
Frequency distribution of calf age (d) at the time of transport. All live calves were transported to calf-raising facilities for a duration of 0.5, 8, 17, or 24 h (10, 830, 1700 or 2500 km, respectively). Bars represent the frequency (n) of calves according to their age (d) relative to calving at the time of transportation.

The frequency distribution of newborn calves relative to their BW and transport duration by parity (1 to 14), gestation length (short, average and extended), birth season (spring, summer, fall and winter), calf sex (female and male), calf breed (dairy and beef-dairy cross), birth farms (1 to 15), and year (2022, 2023, and 2024) are reported in Tables 2 and 3. The frequency distribution of newborn calves relative to parity (1 to 14), gestation length (short, average, and extended), sex (male and female), breed (dairy and beef-dairy cross), birth farm (1 to 15), and birth season, and year by transport duration (0.5, 8, 17, and 24 h) are reported in Table 3. The frequency distribution of newborn calves relative to calf age at transportation is reported in Figure 4.

**Table 2.** Distribution of newborn calves and their birth weights by parity, season, sex, breed, farm, and year

Items	Calves^1^, n	Calf birthweight, kg
**Dam parity**		
** 1**	122,399	26.35
** 2**	92,783	35.58
** 3**	73,666	37.49
** 4**	51,433	38.08
** 5**	27,600	37.94
** 6**	13,890	38.00
** 7**	6,522	37.78
** 8**	2,581	37.58
** 9**	851	36.71
** 10**	260	36.56
** 11**	65	34.99
** 12**	11	38.46
** 13**	2	41.73
** 14**	1	44.90
**Gestation length**		
** 254–269 d**	31,277	26.66
** 270–283 d**	267,057	32.67
** 284–298 d**	93,730	38.96
**Calf birth season**		
** Fall**	85,277	32.24
** Winter**	126,709	34.81
** Spring**	86,632	33.67
** Summer**	93,446	33.50
**Calf sex**		
** Female**	245,901	30.97
** Male**	146,163	38.26
**Calf breed**		
** Dairy**	125,953	27.32
** DB cross**	266,111	36.70
**Farms**		
** 1**	18,257	38.56
** 2**	19,129	31.82
** 3**	14,363	39.46
** 4**	16,829	38.45
** 5**	18,138	36.56
** 6**	17,432	38.86
** 7**	36,654	37.35
** 8**	16,836	36.83
** 9**	21,040	39.15
** 10**	20,668	36.06
** 11**	17,808	39.22
** 12**	142,891	27.13
** 13**	15,008	36.71
** 14**	14,491	39.12
** 15**	2,520	36.04
**Calf birth year**		
** 2022**	166,576	33.74
** 2023**	191,665	33.31
** 2024**	33,823	35.60

1 Calves (*n* = 392,064) born within the south system were transported 0.5 and 8 h (10 and 830, respectively) while calves born within the north system were transported 17 and 24 h (1700 and 2500 km, respectively). The overall birth weight of calves was 28.68 kg for the south system and 38.35 kg for north system

**Table 3.** Distribution of newborn calves relative to parity, sex, breed, farm, and birth season, and year by transport duration (h)

	Calf transport duration^1^, h			
Items	0.5	8	17	24
**Dam parity**				
** 1**	106,269	16,130	–	–
** 2**	15,417	4,141	56,562	16,663
** 3**	572	63	62,966	10,065
** 4**	22	–	44,241	7,170
** 5**	10	17	25,259	2,314
** 6**	16	19	13,337	518
** 7**	29	40	6,227	226
** 8**	35	45	2,383	118
** 9**	25	17	776	33
** 10**	7	7	238	8
** 11**	4	6	53	2
** 12**	1	–	10	–
** 13**	–	–	2	–
** 14**	–	–	1	–
**Gestation length**				
** 254–269 d**	23,457	1,078	5,086	1,656
** 270–283 d**	92,346	16,049	127,420	31,242
** 284–298 d**	6,604	3,358	79,549	4,219
**Birth season**				
** Fall**	25,112	3,408	48,424	8,333
** Winter**	39,098	8,797	67,769	11,045
** Spring**	28,860	3,830	46,516	7,426
** Summer**	29,337	4,450	49,346	10,313
**Calf sex**				
** Female**	109,263	9,801	91,328	35,509
** Male**	13,144	10,684	120,727	1,608
**Calf breed**				
** Dairy**	96,309	–	–	29,644
** DB cross**	26,098	20,485	212,055	7,473
**Farms**				
** 1**	–	–	15,477	2,780
** 2**	–	–	14,421	4,707
** 3**	–	–	11,596	2,767
** 4**	–	–	15,447	1,382
** 5**	–	–	15,180	2,958
** 6**	–	–	14,124	3,308
** 7**	–	–	35,614	1,040
** 8**	–	–	13,143	3,693
** 9**	–	–	17,942	3,098
** 10**	–	–	17,125	3,543
** 11**	–	–	15,122	2,686
** 12**	122,407	20,485	–	–
** 13**	–	–	13,191	1,817
** 14**	–	–	12,357	2,134
** 15**	–	–	1,316	1,204
**Calf birth year**				
** 2022**	51,394	16,987	78,658	19,537
** 2023**	59,772	3,498	112,393	16,002
** 2024**	11,241	–	21,004	1,578

1 Calves (*n* = 392,064) born within the south system were transported 0.5 and 8 h (10 and 830 km, respectively) while calves born within the north system were transported 17 and 24 h (1700 and 2500 km, respectively). Calves were transported to two separate calf-raising facilities with similar housing and environmental conditions, one located in Arizona for replacement heifers transported 0.5 (*n* = 122,407) and 24 h (*n* = 37,117), and another located in New Mexico for DB calves (male and female) transported 8 (*n* = 20,485) and 17 h (*n* = 212,055). Two-deck trailers were used to transport calves 8 or more h while single deck trailers were used to transport calves 0.5 h.

#### Associations of pre-weaning health status, mortality, and growth with transport duration

Differences in least squares means (± SEM; Table 4) for calf birthweight, calving assistance (yes or no), FPT, and calf age (d) at transportation, preweaning diarrhea and pneumonia, mortality upon arrival and at weaning, preweaning ADG and weaning weight relative to transport duration (0.5, 8, 17, and 24 h) were computed using MIXED or GLIMIX procedures of SAS (SAS Institute Inc, 2014). Transport duration was forced in all models as a fixed effect. To perform an analytical control of confounders, other variables offered to the model as fixed effects included FPT, calving assistance, parity, birth season, calf sex, and year. Nonsignificant variables were eliminated from the model one at a time using the Wald statistic backward selection criterion (*P *> 0.15) because of their lack of effect on the outcome variable. Potential confounders were evaluated using a change-in-estimate approach, but no nonsignificant variable produced a ≥ 20% change in the main exposure effect estimate; therefore, only statistically significant variables were retained in the final model. Birth farm and calf-raising facility were included as random effects. The differences in least squares means of the parameters of interest were computed by including the PDIFF option in the LSMEANS statement. The Tukey-Kramer method was used to obtain adjusted individual least squares means for multiple comparisons. Least squares means (±SEM) were reported (Table 4). A *P < *0.05 was considered statistically significant and a *P *≤ 0.10 was considered a tendency to differ.

**Table 4.** Distribution of calves regarding the effect of birthweight, calving assistance, FPT, age at transport, calf mortality upon arrival at calf-raising facility, pre-weaning diarrhea and pneumonia, and calf mortality at weaning, and ADG by transport duration

Items	Calf transport duration^1^, h				
	South system		North system		
	0.5	8	17	24	*P*-value
**Calf birthweight^2^, kg**	28.50 ± 0.95^d^	32.09 ± 0.95^c^	37.41 ± 0.28^a^	33.84 ± 0.69^b^	<0.0001
**Calving assistance^3^, %**	3.48 ± 0.91^bd^	4.21 ± 0.92^ac^	3.61 ± 0.26 ^cd^	4.79 ± 0.28^ab^	<0.0001
**FPT <5.1 g/dL^4^, %**	1.23 ± 0.12^a^	1.22 ± 0.23^a^	0.41 ± 0.12^b^	0.21 ± 0.10^b^	<0.0001
**Calf age at transport, d**	1.63 ± 0.17^d^	6.93 ± 0.17^a^	3.08 ± 0.04^c^	3.47 ± 0.04^b^	<0.0001
**Calf survival and health**					
**Mortality upon arrival^5^, %**	0.015 ± 0.010	0.005 ± 0.011	0.027 ± 0.006	0.009 ± 0.008	0.32
**Diarrhea preweaning^6^, %**	17.15 ± 0.77^a^	3.75 ± 0.77^b^	5.41 ± 0.24^b^	16.40 ± 0.27^a^	<0.0001
**Pneumonia preweaning^7^, %**	7.21 ± 1.49^ab^	1.75 ± 1.49^c^	9.48 ± 0.42^a^	7.64 ± 0.44^b^	<0.0001
**Mortality at weaning^8^, %**	3.65 ± 0.25^a^	1.05 ± 0.26^d^	2.53 ± 0.10^b^	2.08 ± 0.12^c^	<0.0001
**Pre-weaned calf performance^9^**					
**BW at weaning, kg**	67.39 ± 0.96^d^	83.69 ± 0.82^b^	86.34 ± 0.65^a^	80.54 ± 1.12^c^	<0.0001
**Birth to weaning ADG, kg/d**	0.65 ± 0.02^d^	0.86 ± 0.04^a^	0.82 ± 0.02^a^	0.76 ± 0.01^c^	<0.0001

1 Least squares means (±SEM) of variables assessed by calf transport duration of 0.5, 8, 17, or 24 h (10, 830, 1700, or 2500 km, respectively). Calves born within the south system were transported 0.5 and 8 h while calves born in the north system were transported 17 and 24 h. Calves were transported to two separate calf-raising facilities with similar housing and environmental conditions; one located in Arizona for replacement heifers transported 0.5 (*n* = 122,407) and 24 h (*n* = 37,117), and another located in New Mexico for DB calves (male and female) transported 8 (*n* = 20,485) and 17 h (*n* = 212,055). Within a row, least-squares means without a common superscript letter (a–d) differed statistically at P < 0.05.

2 Individual newborn calf birth weights were recorded using a scale at birth. Newborn calves were identified using a numbered ear tag, fed colostrum and their umbilical cords disinfected.

3 Proportion (%) of dams experiencing assisted calving (yes or no). Assisted calving was defined as a dam with a score of ≥2 at calving using a 4-point scale.

4 Failure of passive transfer (FPT) of immunity was assessed in a subset of calves (*n* = 46,377). FPT was defined as total calf serum protein <5.1 g/dL performed at 2–3 d of age (Godden et al., 2019; Lombard et al., 2020). Calves were fed one (0.5 and 8 h) or two colostrum meals (17 and 24 h).

5 Calf mortality upon arrival was defined as a calf that die upon arrival at calf-raising facility following transportation.

6 A case of calf diarrhea was defined as a watery fecal discharge pre-weaning (score 4 using a 4-point scale; Larson et al., 1977).

7 A case of calf pneumonia was defined as the presence of abnormal ocular and nasal discharge, cough, and high rectal temperature (score ≥5 using Wisconsin calf respiratory scoring chart; Buczinski et al., 2015).

8 Calf mortality at weaning was defined as a calf that died upon arrival at calf raising-facility and up to weaning. Calf weaning occurred at 60 ± 3 d of age. Calf-raising facilities: 3.16% for replacements^a^ and 1.85% for DB calves^b^ (^a, b^*P *< 0.0001).

9 In a subset of calves (*n* = 84,449), live body weights were recorded after weaning in groups and then subtracted their individual birthweights to compute the individual average daily gain (ADG) accounting for the calf age (d), which was standardized to 60 d to adjust for age differences

#### Association of calf mortality with transport duration

The differences in calf mortality up to weaning relative to transport duration (0.5, 8, 17, and 24 h; Table 5) were computed using GLIMMIX procedures of SAS (SAS Institute Inc, 2014). Transport duration was forced in all models as a fixed effect. To perform an analytical control of confounders, other variables offered to the model as fixed effects included parity of dams, calf birth season and year, calf sex, and age of calf at transportation. Nonsignificant variables were eliminated from the model one at a time using the Wald statistic backward selection criterion (*P *> 0.15) because of their lack of effect on the outcome variable. Potential confounders were evaluated using a change-in-estimate approach, but no nonsignificant variable produced a ≥ 20% change in the main exposure effect estimate; therefore, only statistically significant variables were retained in the final model. Calf breed, sex and season of birth were not statistically significant; thus, removed from the model. Birth farm and calf-raising facility were included as random effects. The differences in least squares means of the parameters of interest were computed by including the PDIFF option in the LSMEANS statement. The Tukey-Kramer method was used to obtain adjusted individual least squares means for multiple comparisons. Least squares means (±SEM) were reported (Table 5). A *P < *0.05 was considered statistically significant and a *P *≤ 0.10 was considered a tendency to differ.

**Table 5.** Distribution of calf mortality (%) up to weaning relative to parity, gestation length, birth season, FPT, sex, breed, farm, and year by transport distance (h)

	Calf Transport Duration, h^1^				
	South System		North System		
Items	0.5	8	17	24	*P*-value
**Dam parity**					
** 1**	4.75 ± 0.26^a^	0.74 ± 0.21^b^	–	–	<0.0001
** ≥2**	3.41 ± 0.29^a^	0.78 ± 0.34^d^	2.10 ± 0.07^b^	1.79 ± 0.11^c^	<0.0001
**Gestation length**					
** 254–269 d**	5.43 ± 0.59^a^	1.21 ± 0.84^b^	5.21 ± 0.36^a^	3.61 ± 0.56^ab^	0.02
** 270–283 d**	3.19 ± 0.26^a^	1.03 ± 0.28^c^	2.29 ± 0.09^b^	1.83 ± 0.12^c^	<0.0001
** 284–298 d**	3.02 ± 0.25^a^	0.39 ± 0.32^b^	2.64 ± 0.11^a^	2.74 ± 0.24^a^	<0.0001
**Birth season**					
** Fall**	3.41 ± 0.22^a^	1.22 ± 0.33^c^	2.64 ± 0.11^b^	1.60 ± 0.19^c^	<0.0001
** Winter**	2.93 ± 0.37^a^	1.10 ± 0.40^b^	2.53 ± 0.13^a^	1.42 ± 0.18^b^	<0.0001
** Spring**	2.95 ± 0.25^a^	0.09 ± 0.33^d^	1.82 ± 0.11^b^	1.33 ± 0.18^c^	<0.0001
** Summer**	5.01 ± 0.37^a^	0.59 ± 0.46^d^	3.22 ± 0.17^c^	3.77 ± 0.23^b^	<0.0001
**Calf sex**					
** Female**	3.41 ± 0.29^a^	0.97 ± 0.33^c^	2.17 ± 0.11^b^	1.80 ± 0.12^c^	<0.0001
** Male**	2.42 ± 0.29^a^	0.84 ± 0.30^b^	2.80 ± 0.10^a^	3.53 ± 0.38^a^	<0.0001
**Calf breed**					
** Dairy**	3.83 ± 0.38^a^	–	–	1.19 ± 0.38^b^	<0.0001
** DB cross**	2.39 ± 0.27^ab^	0.82 ± 0.29^c^	2.11 ± 0.09^b^	3.22 ± 0.18^a^	<0.0001
** FPT <5.1 g/dL**					
** Yes**	10.94 ± 3.07	6.70 ± 8.2	3.78 ± 2.16	3.41 ± 6.3	0.27
** No**	3.31 ± 0.20^a^	0.52 ± 0.48^d^	2.19 ± 0.10^b^	1.56 ± 0.19^c^	0.0003
**Farms**					
** 1**	–	–	2.19 ± 0.12	1.96 ± 0.29	0.48
** 2**	–	–	1.64 ± 0.10	1.30 ± 0.20	0.34
** 3**	–	–	2.12 ± 0.10	1.95 ± 0.30	0.82
** 4**	–	–	2.10 ± 0.12^b^	3.12 ± 0.40^a^	0.02
** 5**	–	–	1.67 ± 0.10	1.70 ± 0.25	0.91
** 6**	–	–	2.46 ± 0.15	1.94 ± 0.30	0.11
** 7**	–	–	2.36 ± 0.08	2.15 ± 0.47	0.66
** 8**	–	–	1.96 ± 0.12	1.63 ± 0.25	0.24
** 9**	–	–	2.24 ± 0.11^a^	1.63 ± 0.27^b^	0.04
** 10**	–	–	2.22 ± 0.11	1.87 ± 0.26	0.21
** 11**	–	–	2.50	2.20	0.38
** 12**	2.27 ± 0.08	2.67 ± 0.24	–	–	0.75
** 13**	–	–	2.04 ± 0.12	1.45 ± 0.34	0.12
** 14**	–	–	2.13 ± 0.13	2.28 ± 0.33	0.69
** 15**	–	–	1.12 ± 0.29	0.97 ± 0.41	0.70
**Calf birth year**					
** 2022**	3.00 ± 0.25^a^	0.33 ± 0.28^c^	2.18 ± 0.08^b^	1.01 ± 0.13^c^	<0.0001
** 2023**	4.53 ± 0.30^a^	2.73 ± 0.42^b^	2.38 ± 0.09^b^	2.77 ± 0.16^b^	<0.0001
** 2024**	1.35 ± 0.23	–	1.20 ± 0.09	0.80 ± 0.31	0.43

1 Least squares means of calf mortality (%) up to weaning for the interaction of each variable with transport duration was reported. Calves born within the south system were transported 0.5 and 8 h while calves born in the north system were transported 17 and 24 h. Calves were transported in two-deck trailers to two separate calf-raising facilities with similar housing and environment conditions, one located in Arizona for replacement heifers transported 0.5 (*n* = 122,407; single deck trailers) and 24 h (*n* = 37,117) and another located in New Mexico for DB calves (male and female) transported 8 (*n* = 20,485) and 17 h (*n* = 212,055). Different superscript letters within rows for each variable by transport duration differed statistically at *P *< 0.05.

The overall mean calf mortality (%) up to weaning were reported below for the following main effects:

Calf-raising facilities: 3.16% for replacement heifers^a^ and 1.85% for DB calves^b^ (^a, b^*P *< 0.0001).

Calf birth season: 2.70% for Fall^b^, 1.98% for Spring^c^, 3.36% for Summer^a^, and 2.58% for Winter^b^ (^a, b, c^  *P *< 0.0001).

Calf sex: 2.58% female^b^ and 2.84% male^a^ (^a, b^  *P *< 0.0001).

Calf breed: 2.65% for DB cross^b^ and 3.08% for dairy^a^ (^a, b^  *P *< 0.0001).

Gestation length: 3.29% short^a^, 1.93% average^c^, and 2.11% extended^b^ (^a, b, c^  *P *< 0.0001).

FPT (<5.1 mg/dL): 4.15% for Yes^a^ and 2.47% for No^b^ (^a, b^*P *< 0.0001).

Parity: 2.93% for first-calf heifers^a^ and 2.70% for lactation 1^b^ (^a, b^  *P *= 0.03).

Farm: 2.76% for farm 1^abc^, 2.13% for farm 2^d^, 2.73% for farm 3^abc^, 2.82% for farm 4^abc^, 2.28% for farm 5 ^cd^, 2.99% for farm 6^ab^, 2.97% for farm 7^ab^, 2.51% for farm 8^bcd^, 2.77% for farm 9^abc^, 2.78% for farm 10^abc^, 3.05% for farm 11^ab^, 3.16% for farm 12^a^, 2.61% for farm 13^bcd^, 2.79% for farm 14^abc^, and 1.74% for farm 15 ^cd^ (^a, b, c^  *P *< 0.0001).

System: 2.67% for North and 3.15% South^a^ (^a, b^  *P *= 0.13).

Year: 2.33% for 2022^b^, 3.10% for 2023^a^, and 1.50% for 2024^c^ (^a, b, c^  *P *< 0.0001)

#### Association of calf transport duration with calf survival

**Figure 5.**
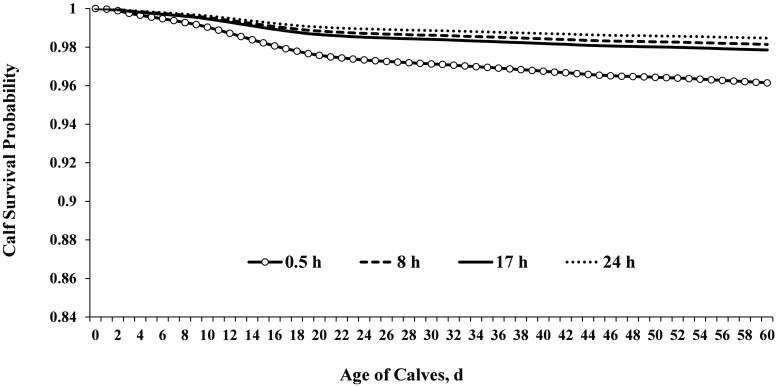
Cumulative calf survival curves for time to mortality (*n* = 392,064) grouped by transport duration. Dairy replacement heifers were transported 0.5 or 24 h (10 or 830 km, respectively) while DB cross calves (male and female) were transported 8 or 17 h (830 or 1700 km, respectively). Calves were transported 0.5, 8, 17, or 24 h (as reference) at a calf-raising facility and calf survival was assessed until weaning (60 ± 3 d). Adjusted hazard ratios (HR; 95% CI) for calf mortality (*P *< 0.0001) were 2.30 (2.14–2.55) for 0.5 h, 0.65 (0.55–0.76) for 8 h, and 1.43 (1.29–1.54) for 17 h.

The association of transport duration with calf survival at calf-raising facility up to weaning were assessed using regression analyses of survival data based on the Cox proportional hazards models using PHREG or PROC LOGISTIC procedures of SAS (SAS Institute Inc, 2014). The overall calf survival was assessed using four groups: 0.5, 8, 17, or 24 h (as reference; Figure 5). The Cox proportional hazard model was used to assess the association of transport duration with survival up to weaning, controlling for the effects of parity of dams, sex (female or male), breed (dairy or DB cross), FPT (yes or no), year, season, calf-raising facility, birth farm, and calf age at transport, if significant. Birth farm was included in the STRATA statement to account for clustering effects. Adjusted hazard ratio (HR) with 95% confidence intervals were reported. Data obtained from SAS output were exported into Excel and plotted to graph the proportion of alive calves over time. A *P *< 0.05 was considered statistically significant and a *P *≤ 0.10 was considered a tendency to differ.

**Figure 6.**
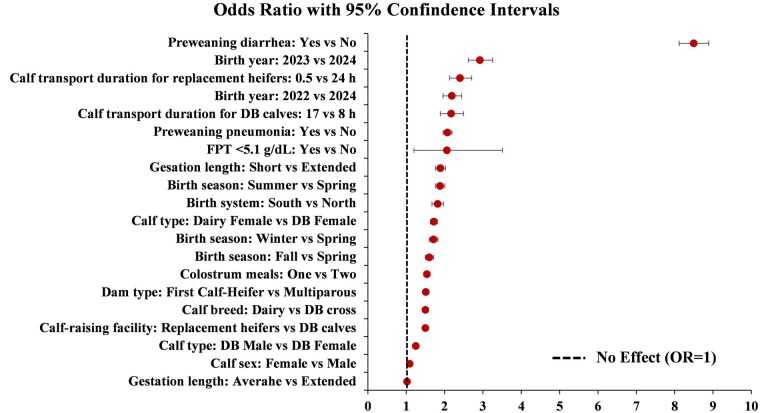
Adjusted odds ratios (OR) and 95% confidence intervals for pre-weaned calf mortality. Calf diarrhea or pneumonia (No as reference), calf birth year (2024 as reference), calf transport duration for replacement heifers (24 h as reference), calf transport duration for BD calves (8 h as reference); calf-raising facility (DB calves as reference), FPT (<5.1 g/dL as reference), colostrum feeding (Two as reference), birth season (Spring as reference), gestation length (Extended as reference), calf type (DB female as reference), calf sex (Male as reference), calf breed (DB cross as reference) and parity (Multiparous as reference).

PROC LOGISTIC was used to assess the association of calf mortality at weaning with the effects of calf disease (diarrhea and pneumonia), calf birth system (south vs north), gestation length (short, average or extended), calf birth season, calf sex (female and male), calf breed (dairy or DB cross), parity of dams, calf transport duration (0.5, 8, 17, or 24 h), calf type (dairy female, DB female, or DB male), and calf birth year (Table 6). To address confounding and collinearity, a total of 6 final models (Model A-F; Table 6) were developed; each tailored to specific subsets of cohort. Model B was designated as the primary inferential model to evaluate the association of transport duration with calf mortality while adjusting for covariates (predictor variables). The remaining models (A, C–F) were developed as contextual models provided to transparently document robustness and illustrate system-level constraints (e.g., structural confounding between transport duration, calf characteristics, calf-raising facility, colostrum meals). Nonsignificant variables were eliminated from the model one at a time using the Wald statistic backward selection criterion (*P *> 0.15) because of their lack of effect on the outcome variable. A change-in-estimate approach was also applied to evaluate potential confounding; however, no nonsignificant variable produced a ≥ 20% change in the main exposure effect estimate. Therefore, only statistically significant variables were retained in the final models. The adjusted odds ratio (OR) with 95% confidence intervals were reported (Figure 6) for preweaned calf mortality at weaning with calf diarrhea or pneumonia (No as reference), calf birth year (2024 as reference), calf transport duration (8 h as reference), calf transport duration for replacement heifers (24 h as reference), calf transport duration for DB calves (8 h as reference), FPT (>5.1 mg/dL as reference), colostrum meals (Two as reference), birth season (Spring as reference), gestation length (Extended as reference), calf type (DB female as reference), calf sex (Male as reference), calf breed (DB as reference), and parity (Multiparous as reference). A *P *< 0.05 was considered statistically significant and a *P *≤ 0.10 was considered a tendency to differ. Furthermore, for each explanatory variable assessed, the individual OR was divided by the sum of all OR and then multiplied by 100 to estimate its relative contribution (%) to the explained outcome (Figure 6).

**Table 6.** Results from a set of multivariable logistic regression models with significant predictors of calf mortality up to weaning at the calf facility after transportation (*n* = 392,064 calves with 9,760 mortalities)

Variable^1^	Description	*n*	Mortality (%)	OR	95% CI	*P*-value	Model
**Calf diarrhea**							A
	No	358,122	1.50	Reference			
	Yes	33,942	11.16	8.49	8.12–8.89	<0.0001	
**Calf pneumonia**							A
	No	353,012	2.23	Reference			
	Yes	39,052	4.82	2.08	1.96–2.19	<0.0001	
**Calf birth system**							A
	North	249,172	2.08	Reference			
	South	142,892	3.19	1.82	1.67–1.97	<0.0001	
**Gestation length**							A
	254–269 d	31,277	4.25	1.89	1.76–2.02	<0.0001	
	270–283 d	267,057	2.35	1.02	0.97–1.07	0.31	
	284–298 d	93,730	2.29	Reference			
**Calf birth season**							A
	Winter	126,709	2.67	1.71	1.60–1.82	<0.0001	
	Spring	86,632	1.72	Reference			
	Summer	93,446	3.26	1.88	1.76–2.00	<0.0001	
	Fall	85,277	2.31	1.59	1.49–1.71	<0.0001	
**Calf sex**							A
	Female	245,901	2.56	1.09	1.04–1.13	<0.0001	
	Male	146,163	2.36	Reference			
**Calf breed**							A
	Dairy	125,053	3.20	1.50	1.45–1.57	<0.0001	
	DB	266,111	2.14	Reference			
**FPT <5.1 g/dL**							A
	No	46,111	2.25	Reference			
	Yes	268	6.34	2.06	1.20–3.51	0.03	
**Colostrum meals**							A
	One	142,892	3.19	1.54	1.48–1.61	<0.0001	
	Two	249,172	2.08	Reference			
**Dam parity**							A
	1	122,399	3.22	1.51	1.45–1.57	<0.0001	
	≥2	269,665	2.16	Reference			
**Calf transport duration**							B
	0.5 h	122,407	3.56	3.60	3.12–4.13	<0.0001	
	8 h	20,485	1.01	Reference			
	17 h	212,055	2.18	2.17	1.88–2.49	<0.0001	
	24 h	37,117	1.55	1.53	1.31–1.80	<0.0001	
**Calf transport duration for replacement heifers**							C
	0.5 h	122,407	3.56	2.34	2.14–2.55	<0.0001	
	24 h	37,117	1.55	Reference			
**Calf transport duration for DB calves**							D
	8 h	212,055	1.01	Reference			
	17 h	20,485	2.18	2.17	1.88–2.49	<0.0001	
**Calf-raising facility**							A
	Replacement heifers	159,524	3.09	1.50	1.44–1.56	<0.0001	
	DB calves	232,540	2.07	Reference			
**Calf type**							E
	Dairy female	125,944	3.20	1.72	1.63–1.81	<0.0001	
	DB male	146,154	2.36	1.25	1.19–1.32	<0.0001	
	DB female	119,957	1.89	Reference			
**Calf birth year**							F
	2022	166,576	2.11	2.19	1.96–2.19	<0.0001	
	2023	191,665	3.04	2.92	2.62–3.25	<0.0001	
	2024	33,823	1.21	Reference			

1 The overall calf mortality upon arrival was 0.015% (58 mortalities out of 392,064 calves). Calf mortality upon arrival at calf-raising facilities was not different (*P *= 0.32) by transport duration. This table provides a summary of significant predictors identified across a set of models, some of which were stratified or built separately to address issues of confounding and collinearity. Not all variables listed were included simultaneously in the same model. Calves were transported to two separate calf-raising facilities with similar housing and environmental conditions; one located in Arizona for replacement heifers transported 0.5 (*n* = 122,407) and 24 h (*n* = 37,117), and another located in New Mexico for DB calves (male and female) transported 8 (*n* = 20,485) and 17 h (*n* = 212,055).

Model A: Calf diarrhea, calf pneumonia, birth system, calf sex, calf breed, dam parity, colostrum meals, gestation length, birth season, and birth year. Full cohort model (*n* = 392,064); all predictors assessed across both systems. No significant multicollinearity was detected among variables.

Model B: Same variables as model A. Full cohort model including transport duration while adjusting for all other covariates. Although no statistical multicollinearity was observed, transport duration was structurally confounded with factors such as calf type, calf-raising facility, birth farm, and colostrum feedings, limiting causal inference.

Model C: Transport duration for replacement heifers (0.5 vs 24 h). Variables included: Transport duration, colostrum meals, dam parity, calf sex, birth season, and birth year. Subset model for replacement heifers only (*n* = 159,524). Stratified by calf type and calf-raising facility to reduce confounding.

Model D: Transport duration for DB calves (8 vs 17 h). Variables included: Transport duration, colostrum meals, dam parity, calf sex, birth season, and birth year. Subset model for dairy-beef (DB) calves only (*n* = 232,540). Stratified to address confounding by calf-raising facility and calf breed.

Model E: Calf-raising facility. Variables included: calf-raising facility, calf type, birth season, and birth year. Full cohort model to explore facility effects, not included simultaneously with transport duration due to collinearity. Calf-raising facility is structurally confounded with transport duration and calf type. Colostrum meals are confounded with transport duration, which in turn is confounded with calf-raising facility and calf type; therefore, colostrum meals were not included.

Model F: Calf type (dairy female, DB male, DB female). Variables included: Birth season, dam parity, and calf-raising facility. Full cohort model. Calf type was included as a proxy for differences in sex and breed, but this variable was also partially confounded with transport duration and facility. Calf type is structural linked to transport duration and colostrum meals; therefore, colostrum meals was not included.

Model designation and interpretation: Model B is the primary inferential model for calf mortality up to weaning, as it evaluates the association of transport duration (0.5, 8, 17, and 24 h) with survival while adjusting for other covariates (predictor variables). Models A and C–F are presented as contextual models to document robustness and system-level constraints (e.g., structural confounding between transport duration, calf characteristics, calf-raising facility, colostrum meals). By transparently presenting the models, this approach ensures that readers recognize the complexity of transport, management, environmental, and biological factors influencing calf survival while avoiding misinterpretation.

#### Association of colostrum meals (one or two) with calf diarrhea

**Figure 7.**
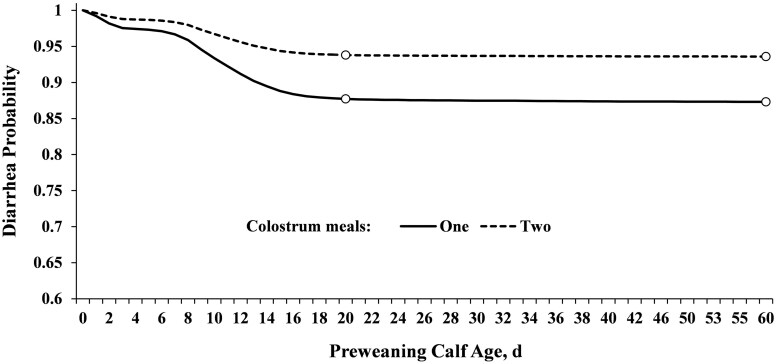
Probability of pre-weaning calf diarrhea in calves fed one or two colostrum meals. Newborn calves were fed one (*n* = 142,892) or two (*n* = 249,172) colostrum meals and calf diarrhea was assessed until weaning (60 ± 3 d). Adjusted HR (95% CI) for the probability of calf diarrhea (*P *< 0.0001) was 2.05 (1.96–2.19) for one compared with two colostrum meals (reference). Calves fed two colostrum meals were ∼50% less likely to develop diarrhea (Open circles) compared to one meal, primarily within the first 20 d (6.2% for two meals and 12.3% for one meal) and less at 60 d of age (6.4% for two meals and 12.7% for two meals).

The association of one or two colostrum meals with preweaning calf diarrhea up to weaning was assessed using regression analyses of survival data based on the Cox proportional hazards models using PHREG procedures of SAS (SAS Institute Inc, 2014). The probability of calf diarrhea over time was assessed for one or two colostrum meals (as reference; Figure 7). The Cox proportional hazard model was used to assess the association of one or two colostrum meals with probability of calf diarrhea up to weaning, controlling for the effects of parity of dams, sex (female or male), breed (dairy or beef cross), year, season, calf-raising facility, and birth farm, if significant. Furthermore, the Cox proportional hazard was used to assess the time to diarrhea or pneumonia by transport duration. Birth farm was included in the STRATA statement to account for clustering effects. Adjusted HR with 95% confidence intervals were reported. Data obtained from SAS output were exported into Excel and plotted to graph the probability of calves with diarrhea over time. A *P *< 0.05 was considered statistically significant and a *P *≤ 0.10 was considered a tendency to differ.

## Results

A total of 2,334 calves were removed from the analyses because they were born from dams with short (<254 d) or extended (>298 d) gestation length. Therefore, a total of 392,064 calves were available for the analyses. About 90% of all dams were HxJ cross and the remaining were 5% Holstein and 5% Jersey. Multiparous cows were housed in the north systems and primiparous cows were housed in the south system (Figure 1). All replacement heifers born in the south and north systems were transported 0.5 h (*n* = 122,407) or 24 h (*n* = 37,117), respectively (Figure 1). All DB cross calves born in the south and north systems were transported 8 h (*n* = 20,485) or 17 h (*n* = 212,055), respectively (Figure 1). The frequency distribution of calves relative to their age at transportation (Figure 4) as well as the demographic distribution of calf birthweights, parity, season, sex, breed, farm and year by transport duration are provided in Tables 1 and 2, respectively.

### Calf birthweight, calving assistance, FPT, and calf age at transport

The overall calf birthweight was 33.69 kg, 4.03% of calves experienced dystocic births, and an overall 0.71% of all calves had FPT (1.27% for calves fed one colostrum meal and 0.38% for calves fed two colostrum meals; *P *< 0.0001). As shown in Table 4, calves born in the north systems (17 and 24 h) were significantly heavier, experienced more assistance at calving and had less FPT compared to those calves born in the south system (0.5 or 8 h; *P *< 0.0001). The overall frequency of calf age at transportation is provided in Figure 4. All newborn calves were transported within 24 h of birth to a nearby collection center or calf-raising facility (Figure 1). Although calf age at transport differed relative to transport duration (*P *< 0.0001), calves born in the north system were transported (17 and 24 h) at about 3 d of age while those born in the south system were transported at ∼1.6 d (0.5 h) and at ∼7 d (8 h; Table 4).

### Calf health status, growth, and survival up to weaning

It is important to note that both calf-raising facilities were designed to be similar in management, housing, and environmental conditions. To address the potential confounding effect of transport duration and calf-rearing facility, all statistical analyses were performed using models that included birth farm and calf-raising facility as random effects. Furthermore, stratified analyses were performed by calf raising-facility (one data set with 0.5 and 24 h, and another with 8 and 17 h) to address issues of confounding and collinearity. The results were consistent with those from the combined model, with no significant change in direction or significance of the key outcomes (calf mortality, health, and ADG); thus, estimates from the combined models were reported. The overall calf mortality during transportation was 0.015% (58 out of 392,064). Preweaning incidence of calf diarrhea and pneumonia significantly differed by transport duration (<0.0001), with the highest diarrhea incidence observed in calves transported for 0.5 and 24 h, and the lowest in those transported 8 or 17 h (Table 4). Pneumonia was highest in calves transported 17 h (9.48%; <0.0001) and lowest in those transported 8 h (1.75%; Table 4), all of which were reared within the same calf-raising facility. The overall calf density (m^2^ per calf) per trailer load was not associated with calf survival upon arrival (*P *= 0.35). Except for the effects of parity, birth season and year (Table 5), the overall calf mortality upon arrival at calf-raising facility was not significantly associated with transport duration (Tables 4 and 5). However, calf mortality at weaning was significantly associated with parity of dams, gestation length, calf birth season, calf sex and breed, farm, and calf birth year (Table 5). Calf mortality up to weaning for each main effects assessed are reported in Table 5. Calves transported 0.5 and 24 h, which were replacement heifers, experienced significantly more diarrhea events compared to calves transported 8 or 17 h (*P *< 0.0001; Table 4). Replacement heifers transported 0.5 or 24 h and beef-dairy cross calves transported 8 or 17 h had similar diarrhea events, regardless of transported distance (Table 4). Calves transported 17 h had more pneumonia events compared to 0.5, 8 or 24 h (Table 4). Calves transported 8 and 17 h (DB cross) had higher ADG and were significantly heavier at weaning compared to 0.5 and 24 h, which were replacement heifers (*P *< 0.0001; Table 4).

**Figure 8.**
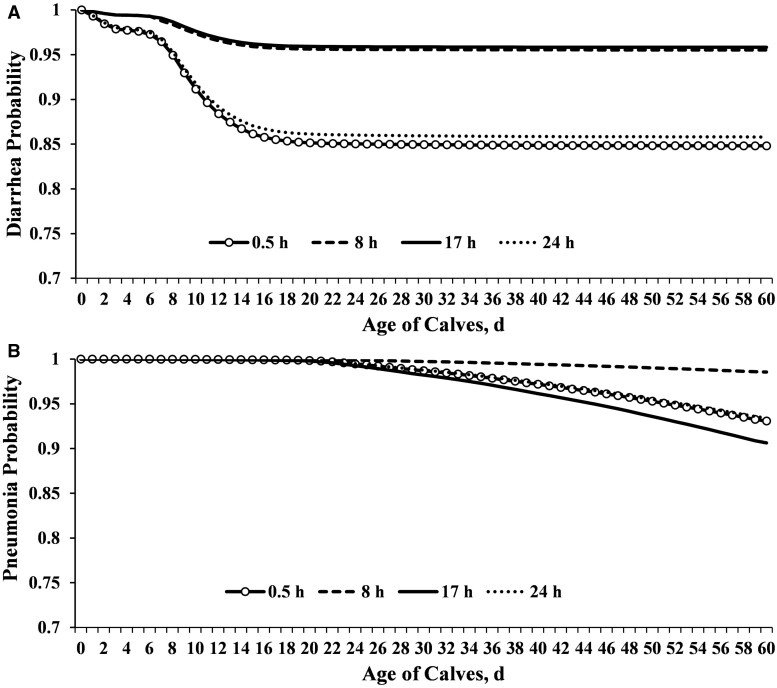
Cumulative survival curves for time to calf diarrhea (A) or pneumonia (B) grouped by transport duration. Dairy replacement heifers were transported 0.5 or 24 h (10 or 830 km, respectively), while DB cross calves (male and female) were transported 8 or 17 h (830 or 1700 km, respectively). Calves were reared at two separate, but similar calf-raising facilities: one housing replacement heifers transported 0.5 or 24 h (as reference) and another housing DB calves transported 8 and 17 h. Calf diarrhea or pneumonia were assessed until weaning (60 ± 3 d). Adjusted HR (95% CI) for calf diarrhea (*P* < 0.0001) were 1.08 (1.04–1.11) for 0.5 h, 0.28 (0.26–0.30) for 8 h, and 0.28 (0.27–0.29) for 17 h. The median time to onset of diarrhea was 10, 11, 10, and 12 for calves transported 0.5, 8, 17, and 24 h, respectively. Adjusted HR (95% CI) for calf pneumonia (*P* < 0.0001) were 1.05 (1.00–1.10) for 0.5 h, 0.21 (0.19–0.24) for 8 h, and 1.44 (1.38–1.50) for 17 h. The median time to onset of pneumonia was 42, 40, 45, and 44 for calves transported 0.5, 8, 17, and 24 h, respectively.

Overall, calf mortality up to weaning was 3.20% for dairy calves and 2.15% for DB calves. Calves transported 0.5 h (replacement heifers born from first-calf heifers) had higher proportion of mortality up to weaning compared to 8, 17 or 24 h (*P *< 0.0001; Table 4). Similarly, the survival analysis revealed that calves transported 0.5 h (replacement heifers born in the south) had higher mortality compared to 8, 17, or 24 h (*P *< 0.0001; Figure 5). Regardless of transport duration, the overall calf mortality at weaning was 2.49% (9,760 dead out of 392,064 calves) for both systems. Calves experiencing FPT had increased mortality up to weaning (*P *< 0.0001), regardless of calf sex (female 3.79% and male 4.68%; *P* = 0.40) or calf breed (DB 3.78% and dairy 4.84%; *P* = 0.81). When assessing the effect of FPT on dairy calves (*n* = 13,976), there were only females available due to the reproductive program with sex-sorted semen for replacements. Regarding the effect of FPT on DB calves (*n* = 32,401), calf mortality up to weaning was 3.53% for females and 4.70% for males (*P *= 0.38). Furthermore, the multivariable regression models revealed the most significant predictors of calf mortality at weaning (Table 6). Calf diarrhea, pneumonia, and birth system (south) were the most influential factors associated with calf mortality risk, contributing with 31.7, 7, and 6.9%, respectively (Table 6). Transport duration was confounded with calf sex (mostly dairy females), calf-raising facility (replacement heifers or DB calves), and parity. There were seasonal variations in calf mortality, with summer and winter contributing the most with 6.9% and 6.1%, respectively (Table 6). Calves born from dams experiencing either short (254–269 d) or extended gestation length (284–298 d) contributed 5.9% to the overall calf mortality risk (Table 6). Overall, FPT (4.8%), female calves (4.6%), and multiparous cows (5.3%) were additional factors associated with calf survival (Table 6). Given that calf survival was one of the primary outcomes of this study, it is important to highlight that calves fed two colostrum meals were ∼50% less likely to develop diarrhea (Figure 7) and ∼54% less likely to die before weaning (Table 6) compared to those fed one meal (Figure 7). The cumulative survival curves for time to calf diarrhea or pneumonia grouped by transport duration are provided in Figure 8. The median time to onset of diarrhea was 10, 11, 10, and 12 d for calves transported 0.5, 8, 17, and 24 h, respectively (Figure 8). The median time to onset of pneumonia was 42, 40, 45, and 44 d for calves transported 0.5, 8, 17, and 24 h, respectively (Figure 8).

## Discussion

The primary findings of the present study are: 1) The overall calf mortality upon arrival at calf-raising facility was 0.015% and did not differ by transport duration; 2) The overall calf mortality at weaning was 2.49% with calves fed two colostrum meals experienced less diarrhea events and had less mortality before weaning; 3) Replacement dairy heifers born in the south experiencing higher mortality at weaning compared to dairy heifers born in the north or DB cross calves; 4) Calves born in the north system were heavier at birth, mostly due to parity and calf sex, experienced more dystocia and less FPT compared to calves born in the south system; 5) Replacement dairy heifers experienced more diarrhea events, regardless of transport duration, compared to DB cross calves while only DB cross calves transported 17 h experienced more pneumonia cases; and 6) transported DB cross calves (8 and 17 h) were heavier at birth and gained more body weight at weaning compared to replacement dairy heifers (0.5 and 24 h).

Although there was a 3-day difference relative to calf age at transportation (north vs south system), all calves were initially transported from maternity to individual hutches within 24 h for south or north system. In the present study, the overall calf mortality risk (upon arrival and at weaning) was much lower than calf mortality reported from previous studies assessing veal calves (Renaud et al., 2018; Cramer et al., 2024). Studies reported preweaned calf mortalities ranging from 0.06% to 0.7% within 24–30 h of transportation (Cave et al., 2005; Thomas and Jordaan, 2013) and up to 7% within the first 21 d after transportation (Winder et al., 2016; Renaud et al., 2018). In the present study, the overall calf mortality up to weaning (60 d of age) was higher for replacement dairy heifers compared to DB cross calves (male or female). The low overall mortality observed in this study may be attributed, at least in part, to factors such as prepartum nutrition of the dams, overall herd management (e.g., newborn care, timely colostrum intake), transport fitness (e.g., logistics), and proper loading and unloading practices. Prepartum nutrition and sound overall management likely supported optimal fetal development and adequate colostrum intake, all of which contribute to improved early calf survival and development. Inference regarding the association between transport duration and calf mortality up to weaning is based on Model B, whereas the additional models (A, C–F) are presented as contextual models to illustrate robustness and to account for system-level constraints (e.g., confounding between transport duration, calf characteristics, calf-raising facility, colostrum meals). Together, these models highlight the complexity of calf transport, management, environmental, and biological factors influencing calf survival.

Calf mortality was not influenced by transport duration as previously reported (Cave et al., 2005; Uetake et al., 2011), but rather by the interaction of calf breed, sex and calf born within systems (north vs south). For instance, female dairy replacements born in the south system experienced more mortality up to weaning compared to DB cross calves (male or female) or female dairy replacements born in the north system (which were exposed to 24 h transportation). Most calves born in the south system were females from primiparous dams inseminated with sex-sorted semen from dairy sires. The observed increased calf mortality up to weaning is likely explained, at least in part, by the summer effect because pregnant dams in the south system were more likely exposed to an extended heat stress period during summer leading to increased preweaned calf mortality (Monteiro et al., 2016; Davidson et al., 2021) compared to those calves born from dams in the north system.

Heat stress has a detrimental effect on placental development and function (Huber et al., 2020; Cattaneo et al., 2022); thus, negatively impacting fetal development and programming. For instance, in utero heat stressed newborn calves have reduced birth weight (Collier et al., 1982), increased FPT (Tao et al., 2012), and subsequent reduced calf growth (Cattaneo et al., 2022) and performance through first lactation (Monteiro et al., 2016). The far-off and prepartum diets (ingredients and nutrient profile) are reported in the present study; however, comparison is not possible because most studies assessing calf mortality after transportation do not report prepartum diets fed to dams. Nutrition management of dams during the last two months of gestation plays a major role for in-utero fetal programming and ultimately affecting survival and performance of their offspring (Abuelo, 2020). For instance, maternal nutrient intake (Grummer et al., 2004) and supply below requirements (e.g., energy, rumen-protected amino acids, fatty acids, trace minerals), due to formulation inconsistencies or factors known to reduce dry matter intake (e.g., environment, inconsistent management, poor housing), have also been associated with decreased calf growth (Alharthi et al., 2017), altered immune responses (Garcia et al., 2014), and reduced survival early in life and during their productive life (Cooke, 2019; Abuelo, 2020). Although genetic selection is critical for calf survival, health and growth, the overall success of a pre-weaning program is largely due to the overall nutrition status of dams, environment (e.g., housing, heat stress), maternity (e.g., colostrum) and post-partum calf feeding and housing management. However, comparing far-off and prepartum diets amongst studies was not possible because most of this critical information is not reported or available.

The overall calf mortality upon arrival was not associated with transport duration; however, calves transported the shortest distance (0.5 h) experienced the highest mortality up to weaning compared with calves transported 24 h, with 8 or 17 h experiencing intermediate mortality risk. The increased calf mortality up to weaning for calves transported 0.5 h (lighter birthweight due to more female dairy calves born from primiparous dams) could be attributed, at least in part, to effect of summer with increased FPT and more cases of diarrhea compared to female dairy calves born in the north system which were transported 24 h (heavier birthweight born from multiparous cows). Although DB cross calves transported 8 h were almost 4–5 d older than any other groups, those calves born in the south system had the greatest FPT (mostly female dairy calves born from first-calf dams with one colostrum feeding) compared to calves born in the north system (dairy and DB cross calves born from multiparous cows with two colostrum feedings). A previous study assessing risk factors for veal calf mortality <21 d of age after transportation was significantly associated with light-weight calves, transported season, and origin of calf supplier (Renaud et al., 2018). Although calf mortality up to weaning was significantly lower for calves transported 24 h (1.55%) compared to 0.5 h (3.56%), calves transported 24 h were exclusively dairy female calves born from multiparous cows in the north system and fed colostrum twice.

Calves experiencing FPT (total serum protein <5.1 g/dL) had increased calf mortality preweaning (Godden et al., 2019); however, in the present study only female dairy calves transported 0.5 h had both increased FPT and mortality risk up to weaning compared to DB cross calves born at the same farm but transported 8 h. Given that calf survival was one of the primary outcomes of this study, it is important to highlight that calves fed two colostrum meals were ∼50% less likely to develop diarrhea and ∼54% less likely to die before weaning compared to those fed one meal. It has been shown that extending or feeding an additional meal of colostrum significantly increased serum IgG concentrations (Hare et al., 2020) and reduced diarrhea (Abuelo et al., 2021). Interestingly, despite that dairy female and DB cross calves were born and managed at the same farm (south system), only female dairy replacements transported 0.5 h had high morbidity (more cases of diarrhea and pneumonia) and mortality risk at weaning (3.56%) compared to DB cross calves transported 8 h (1.01%). Although calf diarrhea and pneumonia differed by transport duration, it was mostly due to calf-raising facility (DB calves transported 8 or 17 h compared to replacement heifers transported 0.5 or 24 h). Differences likely reflect transport duration, calf type, dam parity, and early life care (e.g., colostrum) as replacement heifers (0.5 and 24 h) and DB calves (8 and 17 h) were reared in separate, but similarly equipped, facilities. Both calf-raising facilities used the same standardized health screening and scoring system. However, there is a possibility of inter-observer variability in health assessments. Differences in calf health outcomes may reflect true health status or variation in how the scoring system was applied by different observers. Inter-rater reliability testing was not conducted and represents a limitation of the study.

Despite that DB calves transported 17 h had higher pneumonia (9.48%) compared to 8 h DB calves (1.75%), ADG at weaning was not different for both groups. This observation is consistent with a previous study assessing pneumonia and growth of DB calves (Fernandes et al., 2025). The overall calf mortality up weaning for DB cross calves in the present study was similar to those findings reported by Pereira et al. (2024), but much less than previously reported in the literature for Holstein calves (3.2% during the preweaning period; Lombard et al., 2020). It has been reported that preweaned calf mortality is higher for dairy compared to DB cross calves (Raboisson et al., 2013). Preweaned DB cross calves (beef sires; dairy dams) have been shown to be more resilient to morbidity and mortality compared to dairy calves (Raboisson et al., 2013; Hyde et al., 2020). In a randomized study using 175 experimental units, calves transported 16 h have been shown to increase abnormal fecal and respiratory scores without negatively affecting ADG up to 50 d following transport in surplus calves (Goetz et al., 2023). In the present study, all transported calves were born from the same genetic base cows across multiple farms, operated under the same overall management and nutrition, and reared in two similar calf-raising facilities (one for replacement heifers and another for DB calves). For replacement heifers, preweaning calf health (diarrhea and pneumonia) was unchanged with calves transported 0.5 h having lower ADG up to weaning compared to calves transported 24 h, mostly due to the confounding effect of parity. On the other hand, DB calves transported 8 h experienced less diarrhea and more pneumonia cases compared to calves transported 17 h with same ADG up weaning for both groups. These observed differences between both studies could be attributed, at least in part, to differences in transport logistics or calf health scoring, nutrition program (for both cows and preweaned calves), and limited sample size. The overall reduced mortality preweaning observed in DB cross calves could be due, at least in part, to calf health and survival traits acquired through beef sires as opposed to dairy sires (Basiel and Felix, 2022). Enhancing dairy calf health and survival through genetic selection is attainable because there is more genetic variation than previously forecasted (Lynch et al., 2024; Toghiani et al., 2024).

Gestation length was not associated with calf mortality upon arrival at calf-raising facility; however, gestation length of HxJ dams is known to affect calf survival (Vieira-Neto et al., 2017). Calves born from either short or extended gestation length had increased mortality risk up to weaning compared to those calves born from average gestation length. Parity was not associated with the overall effect of calf mortality upon arrival, but calves born from mature cows had lower preweaned mortality compared to first-calf heifers. Calf mortality upon arrival at calf-raising facility was not associated with farm source, but calf mortality up to weaning was higher for calves born within the south system compared to north system. Although a fair comparison is not possible because only calves born up to March of 2024 were included in the present study, year of calf birth was associated with calf mortality upon arrival as well as calf mortality risk up to weaning. Calf mortality up to weaning was higher for calves born during summer and winter with intermediate calves born in spring and fall. A possible explanation for this observation is that gestation length during the dry period could be reduced by the effect of heat stress during summer (Dahl et al., 2016); leading to increased calf mortality (Monteiro et al., 2016).

Calves born in the northern system were found to be heavier at birth compared to those in the southern system, primarily due to factors such as parity of dams, fed colostrum twice within 24 h, shorter period of heat stress during summer, and calf breed (mostly DB cross); all of which contributed to higher rates of dystocia and lower occurrences of FPT. Despite these differences, all calves were subjected to same transportation protocol, with calves in the south system being moved from maternity to individual hutches within 24 h after birth (only fed colostrum once), while those in the north system were transported at 24 h of age. Notably, differences in calf age at transportation were influenced mainly by calf sex and breed, yet the timing remained consistent within the operational framework of each system. A previous study showed that calves experiencing a dystocic birth had increased calf mortality up to 30 d of age (Lombard et al., 2007); however, calves born in the north system had higher calving assistance with lower mortality risk up to weaning (including female dairy replacement heifers transported 24 h) compared to calves born in the south system (mostly dairy heifers). All calves were subjected to the same transportation protocol where calves should drink four consecutive meals to be fit for transport. A possible explanation is that this protocol could have delayed the transportation of calves born from dystocic births; thus, increasing their chances of survival.

Previous studies have reported that transportation of young dairy calves commonly experience dehydration (Pempek et al., 2017), more cases of diarrhea (Bähler et al., 2012), and respiratory disease (Pardon et al., 2012) upon arrival at calf-raising facilities. In the present study, calf mortality risk upon arrival was not affected by transport duration; however, calf mortality at weaning was higher for female dairy calves transported 0.5 h compared to calves transported 8, 17, or 24 h. Although calf mortality up to weaning is multifactorial in nature, certain variables played a more significant role than others. Calf diarrhea, birth year (2023), pneumonia, and birth system (south) were the most influential factors, contributing with 31.7, 10.2, 7, and 6.9%, respectively, to the overall likelihood of calf mortality at weaning. These factors highlight the importance of proper feeding program, environmental or management factors specific to the year, respiratory infections and geographical conditions (gestations in the south system) to prevent calf mortality preweaning. There were seasonal variations, with summer and winter contributing with 6.9% and 6.1%, respectively, highlighting the importance of environmental temperature and climate negatively impacting calf health and survival. Newborn calves are especially vulnerable to heat and cold stress due to their limited ability to thermoregulate. In regions with extreme seasonal climates, such as those represented in this study, calves are more likely to experience environmental stressors shortly after birth. Heat stress during late gestation can also impair fetal development and compromise passive immunity, making calves more susceptible to disease and mortality. During summer, elevated temperatures and humidity can impair immune function and increase the risk of dehydration, while in winter, prolonged cold exposure may increase energy demands and susceptibility to disease. These unique environmental conditions likely contributed to the observed seasonal variation in calf health and survival.

Calves born from dams experiencing either short (254–269 d) or extended gestation length (284–298 d) contributed 5.9% to the overall calf mortality risk. While contributing less individually, FPT (4.8%), female calves (4.6%) and multiparous cows (5.3%) were additional factors highlighting the complex interaction of genetics, nutrition, and immunity associated with improved calf survival. Although calves born in the south system had more FPT compared to north system (15% vs 2%, respectively), it is important to highlight that both systems had >85% of their newborn calves with excellent serum IgG concentration as reported in the consensus recommendations for US dairy farms (Lombard et al., 2020). Overall, calf mortality risk upon arrival and calf mortality risk at weaning were low; thus, these findings emphasize the importance of implementing practical interventions at the farm level to overcome the negative effect of heat stress on prepartum and fetal programming (primarily for calves born within south system) and improved calf genetics primarily for dairy heifer replacements to improve preweaned calf survival and growth.

As with any retrospective observational study, there are numerous confounders to control for; however, this study is unique due to its consistent management structure and uniform practices across the production system. Although this study is observational in nature, the overall calf mortality upon arrival and up to weaning were low (Lombard et al., 2020); showing that transport duration may not necessarily be associated with increased mortality or morbidity as previously reported (Cave et al., 2005; Uetake et al., 2011) when following well established farm management and fit-for-transport protocols. It is important to highlight that calves were sourced directly from farms, subjected to a standardized transport-fitness protocol, and were not exposed to auction markets or commingling with animals from other sources. These practices likely reduced exposure to additional stressors, pathogens, and variable management, which are common in more fragmented or multi-owner calf supply chains. This relatively controlled system may explain, at least in part, the overall low mortality observed upon arrival and up to weaning, highlighting the importance of consistent management to positively influence calf health and survival.

An important consideration is that calves in the North system experienced an initial short transport from the farm to the collection center prior to shipment to the calf-raising facility (transported 17 or 24 h later). Although this represents an additional transport event, it was followed by at least 48 h conditioning period, allowing for rest, feeding, and health assessment, which may have mitigated the impact of the initial transport stress. In contrast, newborn calves in the South system were transported 0.5 h directly from maternity to calf-raising facility within 24 h of birth. While this additional transport bout in the North could be considered as a potential confounder, our findings suggest that early-life health status and FPT played a more influential role in calf outcomes than transport logistics alone. Although associations were identified, the specific impact of transport duration cannot be determined independently from other factors inherent to each transport system.

One limitation of this observational study is that it is not possible to establish causal relationships between calf transport duration and outcome variables (e.g., survival, morbidity, ADG) because correlation does not always imply causation. Additionally, this study did not control for all potential environmental, genetic, and management factors that may have affected the overall health and performance of preweaned calves. Furthermore, transport duration was confounded with several important factors, including calf source, sex, breed, dam parity, which limits direct comparison across groups. While significant associations were found between calf survival and factors such as diarrhea, pneumonia, FPT, and birth system; it is possible that unmeasured confounders could have influenced these outcomes. Collinearity was assessed prior to model building among predictor variables, without observing evidence of significant multicollinearity, indicating reliable and stable multivariable model estimates. Although absolute comparisons between all four transport durations should be interpreted with caution, including all groups in a unified analysis provides a broader understanding of transport duration effects within a single integrated system with the same genetic base for all cows and operated under the same overall management, ownership and transport logistics. However, transport duration was inherently confounded with several calf- and system-level variables, including birth farm, calf sex, calf breed, dam parity, colostrum feedings, and calf-raising facilities. These structural characteristics limit the ability to isolate the effect of transport duration alone, despite statistical adjustment.

## Conclusions

Our findings showed that transporting newborn calves within the first week of life, following well-established newborn calf management and fit-for-transport protocols, did not affect calf mortality upon arrival at the calf-raising facility. However, calf mortality at weaning did differ by transport duration, mostly due to various confounding effects. The primary factors explaining over 68% of calf mortality at weaning were disease (diarrhea and pneumonia), FPT, transport duration (0.5 or 17 h), birth season (summer), year (2023), and gestation length. For DB cross calves, ADG did not differ by parity or transport duration. Although ADG did differ between dairy female calves transported 24 and 0.5 h, it was primarily due to the confounding effect of parity (female calves born from first-calf heifers versus multiparous cows). While a causal relationship between transport duration and survival or ADG cannot be established, these findings show that key health-related factors early in life play a much larger role in calf mortality at weaning than transport duration. Further randomized controlled studies are needed to establish cause-and-effect relationships, providing a more precise understanding of the effects and improvements in young calf transport practices.

## Abbreviations:

ADG: average daily gain
BW: body weight
DB: dairy-beef calves
DCAD: dietary cation-anion difference
FPT: failure of passive transfer of immunity
HR: adjusted hazard ratio
HxJ: Holstein-Jersey cross dams
OR: adjusted odds ratio
TMR: total mixed ration

## References

[skaf341-B1] Abuelo A. 2020. Symposium review: Late-gestation maternal factors affecting the health and development of dairy calves. J. Dairy Sci. 103(4):3882–3893. 10.3168/jds.2019-17278 32037167

[skaf341-B2] Abuelo A , Cullens F, Hanes A, Brester JL. 2021. Impact of 2 versus 1 colostrum meals on failure of transfer of passive immunity, pre-weaning morbidity and mortality, and performance of dairy calves in a large dairy herd. Animals (Basel) 11(3):782. 10.3390/ani11030782 33799858 PMC8001894

[skaf341-B503] Alharthi A. S. M. , Batistel F., Parys C., Helmbrecht A., Loor J. 2017. Maternal rumen-protected methionine supplementation during late-pregnancy affects calf development and growth during early postnatal life. FASEB J. 31:lb293–lb293. 10.1186/s40104-018-0298-1

[skaf341-B507] Bähler C. , Steiner A., Luginbühl A., Ewy A., Posthaus H., Strabel D., Kaufmann T., Regula G. 2012. Risk factors for death and unwanted early slaughter in Swiss veal calves kept at a specific animal welfare standard. Res. Vet. Sci. 92:162–168. 10.1016/j.rvsc.2010.10.009 21094507

[skaf341-B504] Basiel B. L. , Felix T. L. 2022. Board invited review: Crossbreeding beef × dairy cattle for the modern beef production system. Transl. Anim. Sci. 6:txac025. 10.1093/tas/txac025 35399737 PMC8989152

[skaf341-B3] Buczinski S , Ollivett TL, Dendukuri N. 2015. Bayesian estimation of the accuracy of the calf respiratory scoring chart and ultrasonography for the diagnosis of bovine respiratory disease in pre-weaned dairy calves. Prev. Vet. Med. 119(3–4):227–231. 10.1016/j.prevetmed.2015.02.018 25794838

[skaf341-B4] Cattaneo L , Laporta J, Dahl GE. 2022. Programming effects of late gestation heat stress in dairy cattle. Reprod. Fertil. Dev. 35(2):106–117. 10.1071/RD22209 36592976

[skaf341-B5] Cave JG , Callinan APL, Woonton WK. 2005. Mortalities in bobby calves associated with long distance transport. Aust. Vet. J. 83(1–2):82–84. 10.1111/j.1751-0813.2005.tb12203.x 15971826

[skaf341-B6] Collier RJ , Doelger SG, Head HH, Thatcher WW, Wilcox CJ. 1982. Effects of heat stress during pregnancy on maternal hormone concentrations, calf birth weight and postpartum milk yield of holstein cows. J. Anim. Sci. 54(2):309–319. 10.2527/jas1982.542309x 7076593

[skaf341-B7] Cooke RF. 2019. Effects on animal health and immune function. Vet. Clin. North Am. Food Anim. Pract. 35(2):331–341. 10.1016/j.cvfa.2019.02.00 31103185

[skaf341-B8] Cramer MC , Machuca E, Román-Muñiz IN, Edwards-Callaway LN. 2024. Preliminary exploration of the health and behavior around the time of transportation of beef × dairy calves and holstein bull calves 3 days of age or younger in the Western United States. J. Dairy Sci. 107(4):2454–2464. 10.3168/jds.2023-23886 37939843

[skaf341-B508] Dahl G. E. , Tao S., Monteiro A. P. A. 2016. Effects of late-gestation heat stress on immunity and performance of calves. J. Dairy Sci. 99:3193–3198 26805989 10.3168/jds.2015-9990

[skaf341-B9] Davidson BD , Dado-Senn B, Ouellet V, Dahl GE, Laporta J. 2021. Effect of late-gestation heat stress in nulliparous heifers on postnatal growth, passive transfer of immunoglobulin G, and thermoregulation of their calves. JDS Commun. 2(3):165–169. 10.3168/jdsc.2020-0069 36339508 PMC9623764

[skaf341-B10] England Z.A. , Maggard H.L., Fisher A.D., Roadknight N.W., Pempek J.A. 2023. Condition of bob veal calves on arrival at an abattoir in Ohio. Anim. Welf. 32: e7. 10.1017/awf.2022.8 38487447 PMC10936319

[skaf341-B11] Fernandes ILB et al 2025. The association of lung consolidation and respiratory pathogens identified at weaning on the growth performance of beef-on-dairy calves. J. Dairy Sci. 108(4):3980–3990. 10.3168/jds.2024-25617 39788191

[skaf341-B12] Garcia M et al 2014. Effect of supplementing fat to pregnant nonlactating cows on colostral fatty acid profile and passive immunity of the newborn calf. J. Dairy Sci. 97(1):392–405. 10.3168/jds.2013-7086 24239079

[skaf341-B13] Goetz HM et al 2023. A randomized controlled trial investigating the effect of transport duration and age at transport on surplus dairy calves: Part I. Impact on health and growth. J. Dairy Sci. 106(4):2784–2799. 10.3168/jds.2022-22366 36797186

[skaf341-B14] Godden SM , Lombard JE, Woolums AR. 2019. Colostrum management for dairy calves. Vet. Clin. North Am. Food Anim. Pract. 35(3):535–556. 10.1016/j.cvfa.2019.07.005 31590901 PMC7125574

[skaf341-B15] Grummer RR , Mashek DG, Hayirli A. 2004. Dry matter intake and energy balance in the transition period. Vet. Clin. North Am. Food Anim. Pract. 20(3):447–470. 10.1016/j.cvfa.2004.06.013 15471620

[skaf341-B16] Hare KS et al 2020. Feeding colostrum or a 1:1 colostrum: whole milk mixture for 3 days after birth increases serum immunoglobulin G and apparent immunoglobulin G persistency in holstein bulls. J. Dairy Sci. 103(12):11833–11843. 10.3168/jds.2020-18558 33069413

[skaf341-B17] Huber E et al 2020. Fetal programming in dairy cows: effect of heat stress on progeny fertility and associations with the hypothalamic-pituitary-adrenal axis functions. Anim. Reprod. Sci. 216:106348. 10.1016/j.anireprosci.2020.106348 32414470

[skaf341-B18] Hulbert LE , Moisá SJ. 2016. Stress, immunity, and the management of calves. J. Dairy Sci. 99(4):3199–3216. 10.3168/jds.2015-10198 26805993

[skaf341-B19] Hyde RM et al 2020. Quantitative analysis of calf mortality in Great Britain. J. Dairy Sci. 103(3):2615–2623. 10.3168/jds.2019-17383 31954578

[skaf341-B20] Monteiro APA , Tao S, Thompson IMT, Dahl GE. 2016. In utero heat stress decreases calf survival and performance through the first lactation. J. Dairy Sci. 99(10):8443–8450. 10.3168/jds.2016-11072 27522427

[skaf341-B21] NASEM (National Academies of Sciences, Engineering, and Medicine). 2021. Nutrient Requirements of Dairy Cattle: Eighth Revised Edition. Washington, DC: The National Academies Press. 10.17226/25806. 38386771

[skaf341-B22] Larson LL et al 1977. Guidelines toward more uniformity in measuring and reporting calf experimental data. J. Dairy Sci. 60(6):989–991. 10.3168/jds.S0022-0302(77)83975-1

[skaf341-B23] Lombard JE , Garry FB, Tomlinson SM, Garber LP. 2007. Impacts of dystocia on health and survival of dairy calves. J. Dairy Sci. 90(4):1751–1760. 10.3168/jds.2006-295 17369215

[skaf341-B24] Lombard J et al 2020. Consensus recommendations on calf- and herd-level passive immunity in dairy calves in the United States. J. Dairy Sci. 103(8):7611–7624. 10.3168/jds.2019-1795 32448583

[skaf341-B505] Lynch C. , Schenkel F. S., van Staaveren N., Miglior F., Kelton D., Baes C. F. 2024. Investigating the potential for genetic selection of dairy calf disease traits using management data. J. Dairy Sci. 107:1022–1034. 10.3168/jds.2023-23780 37730178

[skaf341-B506] Pardon B. , De Bleecker K., Hostens M., Callens J., Dewulf J., Deprez P. 2012. Longitudinal study on morbidity and mortality in white veal calves in Belgium. BMC. Vet. Res. 8:26. 10.1186/1746-6148-8-26 22414223 PMC3366893

[skaf341-B25] Pempek J , England Z, Habing G, Niehaus A. 2023. Effect of post-transport oral electrolyte supplementation on behavior, health, and hydration in neonatal calves. J. Dairy Sci. 106(Suppl. 1):115. 10.1093/jas/skae011

[skaf341-B26] Pempek J , Trearchis D, Masterson M, Habing G, Proudfoot K. 2017. Veal calf health on the day of arrival at growers in Ohio. J. Anim. Sci. 95(9):3863–3872. 10.2527/jas2017.1642 28992033

[skaf341-B27] Pereira JMV et al 2024. Association of morbidity, mortality, and average daily gain with transfer of passive immunity in dairy-beef crossbred calves up to 60 days of life. J. Dairy Sci. 107(10):8223–8233. 10.3168/jds.2023-24557 38825104

[skaf341-B28] Raboisson D et al 2013. Perinatal, neonatal, and rearing period mortality of dairy calves and replacement heifers in France. J. Dairy Sci. 96(5):2913–2924. 10.3168/jds.2012-6010 23477819

[skaf341-B29] Renaud DL et al 2018. Risk factors associated with mortality at a milk-fed veal calf facility: a prospective cohort study. J. Dairy Sci. 101(3):2659–2668. 10.3168/jds.2017-13581 29290439

[skaf341-B30] SAS Institute Inc. 2014. SAS/STAT 9.4 User’s Guide. 2nd ed. Cary, NC: SAS Institute Inc..

[skaf341-B31] Schuenemann GM , Nieto I, Bas S, Galvão KN, Workman J. 2011. Assessment of calving progress and reference times for obstetric intervention during dystocia in holstein dairy cows. J. Dairy Sci. 94(11):5494–5501. 10.3168/jds.2011-4436 22032372

[skaf341-B32] Scott K , Kelton DF, Duffield TF, Renaud DL. 2019. Risk factors identified on arrival associated with morbidity and mortality at a grain-fed veal facility: a prospective, single-cohort study. J. Dairy Sci. 102(10):9224–9235. 10.3168/jds.2019-16829 31378492

[skaf341-B501] Shivley C. B. , Lombard J. E., Urie N. J., Weary D. M., von Keyserlingk M. A. G. 2019. Management of preweaned bull calves on dairy operations in the United States. J. Dairy Sci. 102:4489–4497. 10.3168/jds.2018-15100 30852014

[skaf341-B33] Thomas GW , Jordaan P. 2013. Pre-slaughter mortality and post-slaughter wastage in bobby veal calves at a slaughter premises in New Zealand. N. Z. Vet. J. 61(3):127–132. 10.1080/00480169.2012.734374 23181407

[skaf341-B34] Tao S , Monteiro APA, Thompson IM, Hayen MJ, Dahl GE. 2012. Effect of late-gestation maternal heat stress on growth and immune function of dairy calves. J. Dairy Sci. 95(12):7128–7136. 10.3168/jds.2012-5697 23021751

[skaf341-B35] Toghiani S , VanRaden PM, Null DJ, Miles AM, Van Tassell CP. 2024. Validating genomic predictions for economic traits in purebred US dairy heifers. J. Dairy Sci. 107(12):11117–11126. 10.3168/jds.2024-25267 39343196

[skaf341-B36] Uetake K , Tanaka T, Sato S. 2011. Effects of haul distance and stocking density on young suckling calves transported in Japan. Anim. Sci. J. 82:587–590. 10.1111/j.1740-0929.2010.00866.x 21794019

[skaf341-B37] USDA. 2016. Dairy 2014, Dairy cattle management practices in the United States, 2014. USDA–APHIS–VS–CEAH–NAHMS, Fort Collins, CO.

[skaf341-B38] Vieira-Neto A , Galvão KN, Thatcher WW, Santos JEP. 2017. Association among gestation length and health, production, and reproduction in holstein cows and implications for their offspring. J. Dairy Sci. 100(4):3166–3181. 10.3168/jds.2016-11867 28161176

[skaf341-B502] Winder C. B. , Kelton D. F., Duffield T. F. 2016. Mortality risk factors for calves entering a multi-location white veal farm in Ontario, Canada. J. Dairy Sci. 99:10174–10181. 10.3168/jds.2016-11345 27720158

